# Environmental and Health Effects of Benzene Exposure among Egyptian Taxi Drivers

**DOI:** 10.1155/2019/7078024

**Published:** 2019-02-03

**Authors:** Zeinab A. Kasemy, Ghada M. Kamel, Gaafar M. Abdel-Rasoul, Ahmed A. Ismail

**Affiliations:** ^1^Public Health and Community Medicine Department, Faculty of Medicine, Menoufia University, Shebin El-Kom, Egypt; ^2^Biochemistry Department, Faculty of Medicine, Menoufia University, Shebin El-Kom, Egypt; ^3^Industrial Health and Occupational Medicine, Faculty of Medicine, Menoufia University, Shebin El-Kom, Egypt; ^4^Bureau of Health Promotion, Kansas State Department of Health and Environment, 1000 SW Jackson St., Suite 230, Topeka, KS 66612-1274, USA

## Abstract

**Objectives:**

To study environmental exposure to benzene as well as hematological effects of benzene exposure in taxi drivers.

**Background:**

Exposure to benzene has been associated with adverse health effects, including haematopoietic disorders.

**Methods:**

A retrospective cohort study was carried out from the beginning of April 2017, through the end of June 2018, on 280 taxi drivers (the exposed group) and 120 unexposed matched individuals (controls). The tools included a predesigned self-administered questionnaire which contained questions about personal data (e.g., age, educational level, and smoking) and exposure data (e.g., duration and nature of exposure). Investigations involved complete blood count. Environmental studies for light-chain aromatic hydrocarbons (BTEX components: benzene, toluene, ethylbenzene, and xylene) were done.

**Results:**

Mean values (ppm) of ambient concentrations of benzene, toluene, ethylbenzene, and xylene (0.81 ± 0.42, 26.69 ± 0.54, 29.36 ± 2.35, and 25.11 ± 2.06, respectively) in the stations were higher than international permissible levels (*P* < 0.001). On studying the clinical manifestations during the last two weeks, the prevalence of pallor, dizziness, headache, waist and back pain, fatigue, dry throat, and discomfort was significantly higher in taxi drivers (27.1%, 24.3%, 21.4%, 28.6%, 45.7%, 24.3%, and 25.7%, respectively) than among the controls (6.7%, 4.2%, 6.7%, 10%, 10%, 6.7%, 6.7%, and 9.2%, respectively). For chronic diseases, hypertension was the most prevalent chronic disease among the drivers (17.1%) than the controls (5.8%) (*P*=0.002). Regarding self-assessment of health status, 20.0% of taxi drivers reported poor health while 31.4% reported very good health (*P* < 0.001). MCH (pg), TLC (×103/*μ*l), and platelets (×103/*μ*l) were significantly lower among taxi drivers (26.33 ± 2.31, 6.55 ± 1.38, and 189.07 ± 53.25, respectively) (*P*=0.005, <0.001, and <0.001 respectively).

**Conclusion:**

Abnormal hematological findings among taxi drivers were found on exposure to benzene. Health of taxi drivers is generally affected. Setting a clinic for periodic checkup and health education for taxi drivers is highly recommended.

## 1. Introduction

The International Agency for Research on Cancer (IARC) has classified benzene as a human carcinogen [1]. Inside the body, benzene is metabolized inside the liver in several steps by cytochrome P450 2E1, where a variety of ring-opened metabolites and ring-hydroxylated metabolites (e.g., phenols and catechols) are generated [2]. These circulating phenolic compounds can be transported to other organs, e.g., the bone marrow, where they are further oxidized via reactions mediated by peroxidases into their highly reactive quinines [3]. Quinones such as hydroquinone (HQ) and para-benzoquinone (p-BQ) are potent hematotoxic and genotoxic compounds that can be converted by NAD(P)H quinone oxidoreductase (NQO1) back to less toxic hydroxylated metabolites [4]. Exposure to benzene has been associated with adverse health effects, including haematopoietic disorders such as bone marrow deficiency that manifested in decrease in the number of circulating blood cells, anemia, thrombocytopenia, leucopenia, aplastic anemia, and acute myelogenous leukemia in both rodents and humans [5]. However, the mechanisms of benzene-induced hematotoxicity are not totally understood. After reduction of lead content in gasoline since 1995 and use of unleaded gasoline in Egypt by 1998, benzene, toluene, xylene, and oxygenates are the most added additives to gasoline, to improve octane number and decrease exhaust of the engine permitting a more efficient fuel combustion [6]. The most commonly used oxygenates include ethyl tertiary butyl ether (ETBE), methyl tertiary butyl ether (MTBE), ethanol, and methanol [7]. Exposure to toluene and xylene may cause neuropsychological symptoms including fatigue, headache, sleepiness, insomnia, depression, anxiety, difficulty in concentrating or memorizing, drunken feelings, and other cognitive disorders [8]. Around the world and in Egypt, some studies focused their work on the health disorders among fuel supply stations' workers [9, 10] and others studied the health status among taxi drivers [11–13], but in Egypt, there is paucity of research studies done on this field, so this work was performed to study exposure to benzene in taxi drivers from rural and urban areas and study the hematological effects of benzene exposure.

### 1.1. Sample Size

Based on past review of the literature [14], sample size had been calculated at 95% CI and power 80%, and totally, 266 taxi drivers were recruited. The sample had been increased to 300 to carry out pilot study and to avoid the drop out. The final sample size was 280 taxi drivers. About 120 individuals matched for age and sex were recruited.

## 2. Participants and Methods

Menoufia Governorate is an Egyptian governorate with more than 4 million individuals. It has several districts; Shibin El Kom district is one of its largest and overcrowded districts. Being the capital of the governorate, Shibin El Kom district, occupied with more than half million individuals, is the center of all main administrative and educational services. Taxi service is available only in this district. It can be imagined how huge the number of population; the taxi serves in addition to all types of buses, cars, and train. Taxi drivers are working from the early morning to late hours in the night every day to earn their living. They are exposed to benzene and all forms of air pollution for many hours. A retrospective cohort study was carried out from the beginning of April 2017 through the end of June 2018. An approval was obtained from the Committee for Medical Research Ethics in Menoufia Faculty of Medicine. After explanation of the study objectives, an oral consent was taken from all participants. To assess the health status of those drivers, it was required to make situation analysis and choose the overcrowded days especially during the education season. At first 4 overcrowded days were determined to continuously monitor the exposure to BTEX in ambient air during eight-hour work shifts. The drivers were chosen randomly by dividing the day into 3 shifts; from 10 o'clock in the morning to 6 o'clock in the evening was the chosen shift to carry out the study. The overcrowded streets in the city were chosen. Then starting from 10 o'clock, the team was distributed, and it was recommended to choose every other taxi to make the choice be in a random way. About 280 drivers from both rural and urban areas of Shibin El Kom district and 120 unexposed matched individuals from the relatives of the drivers and nondrivers were recruited. All the study participants were subjected to the following.

### 2.1. Questionnaire

A predesigned interview questionnaire was conducted by the authors at work site in rural and urban areas of Shibin El Kom city. After review of the related literature, the authors settled a questionnaire, and it was tested for content validity by a board of specialists in Public Health and Community Medicine Department. Cronbach's alpha was 0.81. A pilot study was carried out on 5 subjects to test feasibility and applicability of the tools, and modifications were done accordingly. The questionnaire included data about demographics (e.g., age and gender), special habits (e.g., smoking and alcohol), and occupational histories (e.g., working hours/day and years of exposure). Also, medical history of neurological and hematological manifestation and past history of diseases (e.g., neurological, psychiatric, blood, renal, and diabetes mellitus) were taken. Data were collected from the beginning of the day at 9 a.m., and less crowded days were selected in order to have time to interview the drivers with good temper.

### 2.2. Complete Blood Count (CBC)

Two ml of blood was withdrawn by venipuncture using a sterile plastic syringe. This sample of blood was then delivered into a sterile plastic tube containing EDTA (disodium ethylenediaminetetraacetate) as an anticoagulant, then introduced into automatized counter (Sysmex K 1000) which directly gives Hb concentration, WBCs, RBCs, and platelets counts.

### 2.3. Environmental Measurement (Monitoring of BTEX in Ambient Air by Gas Chromatography)

The exposure to BTEX in ambient air was continuously monitored during eight-hour work shifts inside 120 taxis with ensured commitment to work in the overcrowded urban areas as some rural drivers announced that they finish their work early as their homes were away from the city. Ambient air samples were collected inside the taxi by active sampling with a flow rate 100 ml/min using SKC battery-operated air sampling pump model PCXR4. Activated charcoal cartridges were used to collect samples. At each taxi, two samples were collected at two different days. The contents of each cartridge were placed in a separate vial which was sealed and placed in a cooled path for 20 min and then left for 1 hour at 10°C. The samples were extracted with carbon disulphide (CS_2_), and the sample solution was then analyzed by gas chromatography. VF-5MS column (30 m length, 0.25 mm internal diameter, and 0.25 Pm film thickness) was used for chemical analysis. At 40°C for 3 min, the column oven was initially programmed followed by a heating rate of 15°C·min^−1^ ramp to a final temperature of 200°C. The final temperature was held for 6 min. The BTEX calibration was done using a standard solution (Supelco EPA TO-1 Mix 1A). To build the calibration curve, solutions with concentrations ranging from 0.1 to 4.0 ng·PL^−1^ were used. Correlation coefficients were always above 0.99. The quantification limit calculated for each BTEX was (20 pg·PL^−1^), in relation to a concentration of 1.0 Pg·m^−3^ in the atmosphere. Under the supervision of qualified professor from air pollution department, at the national research center, Cairo, Egypt, all these steps were carried out.

### 2.4. Data Management

Results were collected, tabulated, and statistically analyzed by IBM personal computer and statistical package SPSS version 20 (SPSS Inc., Chicago, USA). Two types of statistics were done: descriptive statistics (e.g., percentage (%), mean (*X*), and standard deviation (SD)) and analytic statistics (e.g., Student's *t-*test was used for comparison of the means of continuous quantitative parametric variables and *Z* test was used for comparison between proportions). Statistical significance was accepted for *P* < 0.05.

## 3. Results

**Figure 1:**
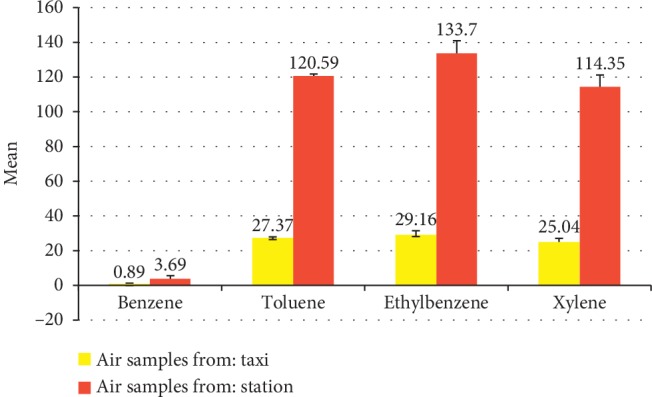
Ambient concentrations of benzene (ppm), toluene, ethylbenzene, and xylene in taxi versus station results.

Ambient concentrations of benzene (ppm), toluene, ethyl benzene, and xylene (0.89 ± 0.42, 27.37 ± 6.59, 29.16 ± 2.50, and 25.04 ± 2.17, respectively) were significantly lower than that measured by Abdel-Rasoul et al. [10] (3.69 ± 1.88, 120.59 ± 1.17, 133.70 ± 7.20, and 114.35 ± 6.86 ppm, respectively) which already exceed the corresponding local and international safety guidelines for these elements (*P* < 0.001) (Table 1 and Figure 1). Regarding the general characteristics of the studied groups, there were no significant differences regarding age, marital status, or income. Regarding smoking, it was significantly higher among drivers (70%) than among the nonexposed group (55.8%) (*P*=0.006) (Table 2). Regarding assessment of health status of the drivers and unexposed group, the prevalence of pallor, dizziness, headache, waist and back pain, fatigue, dry throat, and discomfort was significantly higher in taxi drivers (27.1%, 24.3%, 21.4%, 28.6%, 45.7%, 24.3%, and 25.7%, respectively) than among the controls (6.7%, 4.2%, 6.7%, 10%, 10%, 6.7%, 6.7%, and 9.2%, respectively). For chronic diseases, hypertension was the most prevalent chronic disease among the drivers (17.1%) than the controls (8.3%) (*P*=0.002). Waist and back pain in the last 12 months found to be 54.3% among drivers versus 10% among the unexposed group. Regarding self-assessment of health status, 20.0% of taxi drivers reported poor health versus 0% among the unexposed group while 31.4% of taxi drivers reported very good health versus 70.0% among the unexposed group (*P* < 0.001). There was no significant difference among the studied groups regarding periodic checkup or receiving health education before (*P*=0.922 and 0.875, respectively) (Table 3). Regarding the hematological effects, MCH (pg), TLC (×10^3^/*μ*l), and platelets (×10^3^/*μ*l) were significantly lower among taxi drivers (26.77 ± 3.58, 6.90 ± 1.49, and 204.60 ± 56.49, respectively) than among controls (27.61 ± 2.28, 8.57 ± 1.91, and 266.06 ± 52.87, respectively) (*P*=0.005, <0.001, and <0.001, respectively) while MCHC (gm/dl) was significantly higher among taxi drivers (33.90 ± 1.86) than among the unexposed group (31.36 ± 0.75) (*P* < 0.001) (Table 4).

## 4. Discussion

A higher level of benzene than the permissible level of the Egyptian Ministry of Trade and Industry, Law 4 Decree 1095 (1.56 ppm) [15], was reported in the stations [10]. In the present study, ambient concentrations of benzene (ppm), toluene, ethylbenzene, and xylene were significantly lower than that measured by Abdel-Rasoul et al. [10]. This is in agreement with Bahrami et al. [16] who found that the mean concentration of benzene in breathing zones of petrol station workers was 2-3 times higher than that of the drivers and also 3 times greater than the threshold level (0.5 ppm) as recommended by the American Conference of Governmental Industrial Hygienists (ACGIH). Despite the levels were less than the threshold level, the mean concentration of benzene in the breathing zone of drivers was greater than the reported studies from Asia, Australia, and America [14, 17–20]. Regarding self-assessment of health status, 20.0% of taxi drivers reported poor health versus 0% among the unexposed group while 31.4% of taxi drivers reported good and very good health versus 70% among the unexposed group. This is in agreement with the findings of Yang et al. [14] who found that the prevalence rate of hypertension among taxi drivers was 18.2%, whereas the overall prevalence rate of hypertension in Chinese adults was 18.1% in 2004. Decreased physical activity, being in a stressful job, hard work, and normal sleep disruption may attribute to and might explain the higher prevalence of hypertension among taxi drivers. Regarding waist and back pain, our study revealed the prevalence to be 54.3% in the last 12 months. This is in agreement with the findings of Wang et al. [11] who found the prevalence to be 54% but it is lower than that found by Burgel and Elshatarat [12] who reported low back pain to be 67%. The present data clearly showed reduction in MCH (pg), WBCs, and platelets with no significant difference regarding RBcs, Hb, HCT, and MCV. This result is in agreement with the findings of Avogbe et al. [25] who reported decreased WBCs counts in taxi drivers compared to unexposed controls. Also RBC counts remained unchanged between village inhabitants and exposed MBTD. This result is consistent with many studies, which reported the same at benzene exposure levels ranging from 1.9–14.8 ppm [26] and 0.005–5.3 ppm [27]. However, others reported decreased RBC counts at benzene levels ranging from 0.14–2.08 ppm [28] or found declined RBC in workers with heavy exposure to benzene (maximum level of 34 ppm) [29]. 

## 5. Conclusion

Abnormal hematological findings were found among taxi drivers exposed to high levels of benzene, toluene, ethylbenzene, and xylene concentrations that were more than the permissible levels. Exposed taxi drivers showed a variety of symptoms that related to different systems, e.g., neurological, respiratory, gastrointestinal, cardiovascular, and endocrine as well as significant changes in the blood components. Preventive and screening measures are required to reduce the burden of environmental exposure among taxi drivers. Establishing a clinic for periodic checkup and early detection of symptoms and hematological changes in areas that are easily approached by taxi drivers is highly recommended. In addition, health education programs can be introduced for drivers to increase the taxi drivers' level of awareness of the drawbacks of exposure to those environmental contaminants. Furthermore, employing of gas sensors, specific to the detected gases, is an effective solution to detect gas levels when they exceed the permissible levels.

## Figures and Tables

**Table 1:** Ambient concentrations of benzene (ppm), toluene, ethylbenzene, and xylene in taxi versus station results.

	Air samples		Test	*P* value
	Taxi (no. = 120)	Station^#^ (no. = 40)		
	$\bar{X}\pm \text{SD}$	$\bar{X}\pm \text{SD}$		
Benzene (ppm)	0.89 ± 0.42	3.69 ± 1.88	Mann–Whitney = 8.26	<0.001^*∗*^
Toluene (ppm)	27.37 ± 6.59	120.59 ± 1.17	*t* = 147.93	<0.001^*∗*^
Ethylbenzene (ppm)	29.16 ± 2.50	133.70 ± 7.20	*t* = 89.97	<0.001^*∗*^
Xylene (ppm)	25.04 ± 2.17	114.35 ± 6.86	*t* = 81.01	<0.001^*∗*^

^*∗*^Significant; ^#^Abdel-Rasoul et al. [10].

**Table 2:** Characteristics of drivers and unexposed group.

	Drivers (no. = 280)		Non drivers (no. = 120)		Test of sig.	*P* value
	No.	%	No.	%		
Age (Y), $\bar{X}\pm \text{SD}$	42.80 ± 9.69		43.86 ± 6.74		*t* = 1.26	0.208

Residence						
Urban	190	67.9	90	75.0	*χ* ^2^ 2.04	0.153
Rural	90	32.1	30	25.0		

Education						
Basic	56	20.0	36	30.0	*χ* ^2^ 7.21	0.027
Secondary	200	71.4	80	66.7		
University	24	8.6	4	3.3		

Income						
Enough	132	47.1	58	48.3	0.04	0.827
Not enough	148	52.9	62	51.7		

Marital status						
Single	100	35.7	40	33.3	0.20	0.647
Married	180	64.3	80	66.7		

Smoking						
Smokers	196	70.0	67	55.8	*χ* ^2^ 7.48	0.006^*∗*^
Nonsmokers	84	30.0	53	44.2		

Duration of driving work (years)						
10	40	14.3	—		—	—
≥10	240	85.7				

Driving work hours/day						
<8	26	9.0	—		—	—
≥8	264	91.0				

^*∗*^Significant.

**Table 3:** Health status among the studied groups.

	Drivers (no. = 280)		Unexposed controls (no. = 120)		*Z* test	*P* value
		%		%		
Pallor	76	27.1	8	6.7	4.46	<0.001^*∗*^
Palpitation	60	21.4	6	5.0	3.90	<0.001^*∗*^
Dizziness	68	24.3	5	4.2	4.63	<0.001^*∗*^
Headache	60	21.4	8	6.7	3.44	<0.001^*∗*^
Waist and back pain	152	54.3	12	10.0	8.14	<0.001^*∗*^
Fatigue	128	45.7	12	10.0	6.75	<0.001^*∗*^
Dry throat	68	24.3	8	6.7	3.97	<0.001^*∗*^
Insomnia	40	14.3	9	7.5	1.73	0.082
Anxiety	52	18.6	9	7.5	2.68	0.007^*∗*^
Lack of concentration	72	25.7	12	10.0	3.40	<0.001^*∗*^
Dreaminess	44	15.7	10	8.3	1.83	0.067
Dyspepsia	48	17.1	10	8.3	2.14	0.032^*∗*^
Constipation or diarrhea	16	5.7	4	3.3	0.76	0.447
Frequent micturition	16	5.7	4	3.3	0.76	0.447
Cough and expectoration	36	12.9	6	5.0	2.18	0.029^*∗*^
Chest congestion or asthma	16	5.7	0	0.0	2.39	0.016^*∗*^
Discomfort in last 2 weeks	72	25.7	11	9.2	3.59	<0.001^*∗*^
DM	28	10.0	6	5.0	1.45	0.147
Hypertension	48	17.1	7	5.8	2.97	0.002^*∗*^
Periodic checkup	24	8.6	10	8.3	0.10	0.922
Health evaluation						
Good or very good	88	31.4	84	70.0	7.04	<0.001^*∗*^
Fair	136	48.6	36	30.0	7.0	<0.001^*∗*^
Poor	56	20.0	0	0.0	5.13	<0.001^*∗*^
Receiving health education before	16	5.7	8	6.7	0.16	0.875

^*∗*^Significant.

**Table 4:** Complete blood count of the studied groups.

	Drivers (no. = 280)	Unexposed controls (no. = 120)	*t*-test	*P* value
	$\bar{X}\pm \text{SD}$	$\bar{X}\pm \text{SD}$		
Hemoglobin (gm/dl)	13.69 ± 1.95	13.99 ± 1.63	1.51	0.131
RBCs (×10^6^/*μ*l)	4.86 ± 0.56	4.93 ± 0.52	1.22	0.222
HCT (%)	39.38 ± 6.68	40.44 ± 4.60	1.58	0.113
MCV (fl)	82.43 ± 7.42	83.93 ± 7.21	1.87	0.062
MCH (pg)	26.77 ± 3.58	27.61 ± 2.25	2.82	0.005^*∗*^
MCHC (gm/dl)	33.90 ± 1.86	31.36 ± 0.75	19.34	<0.001^*∗*^
TLC (×10^3^/*μ*l)	6.90 ± 1.49	8.57 ± 1.91	8.52	<0.001^*∗*^
Platelets (×10^3^/*μ*l)	204.60 ± 56.49	266.06 ± 52.87	10.16	<0.001^*∗*^

## Data Availability

The data used to support the findings of this study are available from the corresponding author upon request.
